# Long -term persistence of antibodies against varicella in fully immunized healthcare workers: an Italian retrospective cohort study

**DOI:** 10.1186/s12879-021-06180-x

**Published:** 2021-05-25

**Authors:** Francesco Paolo Bianchi, Silvio Tafuri, Angela Maria Vittoria Larocca, Cinzia Annatea Germinario, Pasquale Stefanizzi

**Affiliations:** 1 Department of Biomedical Science and Human Oncology, Aldo Moro University of Bari, Piazza Giulio Cesare 11, 70124 Bari, Italy; 2 Hygiene Department, Bari Policlinico General Hospital, Bari, Italy

**Keywords:** Varicella zoster virus, Third varicella dose, Duration of immunization, Healthcare workers, Long-term immunogenicity, Chickenpox

## Abstract

**Background:**

Chickenpox is a highly contagious disease caused by the varicella zoster virus (VZV), and in infants, adolescents, adults, pregnant women, and the immunocompromised it can be serious. The best way to prevent chickenpox is immunization with the varicella vaccine. Protective levels of antibodies induced by the varicella vaccine decline over time, but there is currently no formal recommendation for testing anti-varicella zoster virus (VZV) IgG levels in immunized healthcare workers (HCWs).

**Methods:**

The aims of this study were to evaluate the seroprevalence of circulating anti-VZV IgG in a sample a sample of students and residents of the medical school of the University of Bari, the long-term immunogenicity of the varicella vaccine, and the effectiveness of a strategy consisting of a third vaccine booster dose. The study population was screened as part of a biological risk assessment conducted between April 2014 and October 2020. A strategy for the management of non-responders was also examined.

**Results:**

The 182 students and residents included in the study had a documented history of immunization (two doses of varicella vaccine). The absence of anti-VZV IgG was determined in 34% (62/182; 95%CI = 27.2–41.4%), with serosusceptibility more common among males than females (*p* < 0.05). After a third varicella dose, seroconversion was achieved in 100% of this previously seronegative group. No serious adverse events were recorded.

**Conclusions:**

One-third of the study population immunized against VZV lacked a protective antibody titer, but a third dose of vaccine restored protection. Since it is highly unlikely that VZV will be eliminated in the immediate future, the loss of immunity in a substantial portion of the population implies a risk of varicella outbreaks in the coming years. Screening for varicella immunity in routine assessments of the biological risk of medical students and HCWs may help to prevent nosocomial VZV infections.

## Background

Chickenpox is a highly contagious disease caused by the varicella zoster virus (VZV), and in infants, adolescents, adults, pregnant women, and the immunocompromised it can be serious [1]. VZV causes a systemic infection that typically results in lifetime immunity [2] but endogenous reactivation from latency can cause herpes zoster (HZ) [3]. The best way to prevent chickenpox is immunization with the varicella vaccine, available both in monovalent form and in combination with the measles, mumps and rubella (MMRV) vaccine [4]. In the US, since the introduction of the vaccine, > 3.5 million cases of varicella, 9000 hospitalizations, and 100 deaths have been prevented [5]. Furthermore, there was a 17% decline in the rate of HZ among children [6].

At the time of the writing of this study, CDC provides that youngsters receive two doses of varicella vaccine, specifically the first one at 12–15 months and the second at 4–6 years [7]. Moreover, not immunized teenagers and adults should get two doses of the MMRV/varicella vaccine from 4 to 8 weeks apart [7].

Pre-licensure data show that one dose of vaccine prevent any manifestation of varicella in 82% of cases and ˜100% successful against the most serious complications of the disease; two doses prevent any manifestation of varicella in 98% of cases and 100% successful against the most serious complications of the disease [8]. Post-licensure evidences show that two doses of vaccine are ˜92% effective versus all manifestations of disease [8]. The seroconversion rate after two doses is estimated to be > 95% [2].

Since the introduction of global mass vaccination, the varicella vaccine has shown a high level of safety, cost-savings, and efficacy [2, 9, 10]. However, according to the CDC, the duration of protection against varicella following vaccination is unclear. Live vaccines generally provide long-lasting immunity, but thus far only few studies have shown that vaccination against varicella confers protection for almost 10 years thereafter [8].

Since 2003, Italy’s National Immunization Plan has recommended two doses of varicella vaccine, in accordance with CDC guidelines [7]. In 2017, varicella vaccination became compulsory in Italy, mandated by the Italian Ministry of Health, Decree-Law n. 73/2017 [11]. Currently, the Italian National Immunization Plan recommends the administration of either the monovalent varicella vaccine or the tetravalent MMRV vaccine [12]. Despite these measures, the proportion of susceptible Italian adults remains high. For example, in two very recent studies of Italian healthcare workers (HCWs), including those whose vaccination status was unknown, VZV susceptibility was in the range of 6.7–12% [13, 14].

The aims of this study were to evaluate the seroprevalence of circulating anti-VZV IgG in a sample of young adult HCWs, the long-term immunogenicity of the varicella vaccine, and the effectiveness of a strategy consisting of a third vaccine booster dose administered to previously immunized adults without detectable IgG against VZV. The study was carried out in Apulia (southern Italy, ˜4,000,000 inhabitants), a region where previous surveys found a relatively high (7–12%) prevalence of adults susceptible to VZV [15, 16] and where varicella outbreaks have been recorded [17].

## Methods

Since 2010, the Italian Ministry of Health has required that medical schools and university hospitals apply the same procedures mandated by Italian law for the occupational health and safety of HCWs to medical students and residents as well [18]. In response, in April 2014 the Hygiene Department of the Bari Policlinico University Hospital implemented a biological risk prevention program for students and residents of the medical school of the University of Bari. As part of the program, VZV susceptibility/immunity was assessed.

This study was designed as a retrospective cohort study with a study population comprising Apulian students and residents of the medical school of the University of Bari who underwent a biological risk assessment between April 2014 and October 2020. The study exclusion criteria were absence of an available vaccination history, no history of vaccination or vaccinated with a single dose or ≥ 3 doses of varicella vaccine at baseline, and a history of varicella infection. Inclusion criteria were vaccination with two doses of varicella vaccine (vaccine basal routine), either monovalent or tetravalent, prior to study enrollment.

The vaccination status of each participant was confirmed using the Regional Immunization Database (GIAVA) [18], a computerized vaccination registry that contains the vaccination history of every Apulian inhabitant. A 5-mL serum sample was collected from each enrollee to assess his/her VZV immunity/susceptibility status and was tested by chemiluminescence using LIAISON® VZV IgG, a semi-quantitative method performed with a standardized commercial kit (Diasorin). An anti-varicella IgG titer > 150 mIU/mL is defined as seroprotective [19].

Tested participants with a non-protective IgG titer received a third booster dose of monovalent varicella vaccine, administered subcutaneously in the deltoid muscle, consistent with the protocols applied in some US medical schools [20]. Participants with equivocal tests were retested and, if the result was still equivocal, they were classified as seronegative. A further blood test was performed 20–25 days after vaccination to re-measure IgG titers; a value that exceeded the cut-off was considered to indicate seroconversion. If the titer was still negative, another vaccine dose (28 days after the first booster) was administered and after another 20–25 days the IgG titer was again measured. Students/residents who were still seronegative were definitively classified as “non-responders.” Anyone who received a booster dose was followed-up for 1-month to monitor the possible occurrence of adverse effects.

Information on patient identification, sex, age at study enrollment, dates of routine varicella vaccine administration, VZV IgG titer, date of first booster dose, IgG titer after the first booster, date of the second booster dose, and IgG titer after the second booster were collected. The data were imputed in a database created using Office Excel software and analyzed using STATA MP16 software (StataCorp. 2019. Stata Statistical Software: Release 16. College Station, TX: StataCorp LLC) [21].

Continuous variables are expressed as the mean ± standard deviation and range, categorical variables as proportions, with the 95% confidence interval (95%CI), when appropriate. Skewness and kurtosis tests were used to evaluate the normality of the continuous variables, but any of them were normally distributed or normalizable. The Wilcoxon’s rank sum test (not parametric) was used to compare continuous variables between the sexes and the Wilcoxon’s signed-rank test (not parametric) was used to compare them as a function of the evaluation time. Chi-squared and Fisher’s exact tests were used to compare proportions [21].

The determinants of seropositivity at enrollment were assessed in a multivariate logistic regression analysis that included sex (male vs. female), age at enrollment (years), the presence of one or more chronic diseases (yes/no), age at the time of the first vaccination of the basal routine (years), and age at the time of the second vaccination of the basal routine (years). The adjusted odds ratio (aOR) was calculated with the 95%CI. The Hosmer-Lemeshow test was used to evaluate the goodness-of-fit of the multivariate logistic regression model [21].

Protective antibody survival (PAS), defined as the time from the second dose of routine varicella vaccine until the evaluation of the antibody titer, was evaluated using Kaplan-Meier curves, and the differences between sexes using the log-rank test. The median PAS time was estimated and the incidence of the loss of seroprotection per person-year was calculated together with the incidence rate ratio (IRR). In the latter, the incidence in females served as the denominator and that in males as the numerator.

A multivariate Cox semiparametric regression was used to evaluate the determinants of PAS; the risk predictors were as follows: sex (male vs. female), age at enrollment (years), presence of one or more chronic diseases (yes/no), age at the first dose of the routine vaccine (years), and age at the second dose of the routine vaccine (years). The adjusted hazard ratio (aHR) was calculated along with the 95%CI. The Schoenfeld and scaled Schoenfeld residuals tests were used to evaluate the proportionality assumption of the multivariate Cox semiparametric regression model; if one of the predictors was not proportional, the regression model was stratified on the non-proportional predictor. The Gronnesby and Borgan test was used to evaluate the goodness-of-fit of the model.

For all tests, a two-sided *p*-value< 0.05 was considered to indicate statistical significance.

## Results

From April 2014 to October 2020, 6105 medical students and residents participated in the biological risk assessment program. According to the vaccination certificates, available for 5469/6105 (89.6%) participants, 182/5469 (3.3%) received two doses of anti-varicella vaccine. Among the latter, 124 (68.1%) were female; the average age at enrollment was 20.4 ± 2.5 years (range = 18.0–38.0) and did not significantly differ between females (20.6 ± 2.7; range = 18.0–38.0) and males (20.2 ± 2.1; range = 18.0–26.0; *p* = 0.194). At least one chronic disease was reported in 68 of the 182 (37.4%) participants, without a difference between sexes (females: 47/124; 37.9%; males: 21/58; 36.2%; *p* = 0.826). None of the participants had a history of varicella. Average age at dose 1 was equal to 11.3 ± 4.2 (range = 1–32), average age at dose 2 was equal to 14.2 ± 3.2 (range = 9–32).

The 182 study enrollees with a complete basal vaccination routine (two doses) were tested for their titers of anti-varicella IgG. A protective titer was determined in over half (120/182; 65.9%; 95%CI = 58.6–72.9%) and the difference between females (*n* = 91/124; 73.4%; 95%CI = 64.7–80.9%) and males (*n* = 29/58; 50.0%; 95%CI = 36.6–63.4%) was statistically significant (*p* = 0.002). The overall geometric mean anti-varicella IgG titer was 304.5 (95%CI = 250.1–370.8) and also differed significantly between females (367.6; 95%CI = 292.8–461.5) and males (203.7; 95%CI = 140.6–295.0; *p* = 0.006). The geometric mean neutralization titer (GMT) among the seroprotected was 657.1 (95%CI = 556.9–775.4), compared with 68.7 (95%CI = 59.4–79.5) in the non-seroprotected (*p* < 0.0001).

A booster dose was administered to 44 of the 62 (71.0%) seronegatives. Within this group 34 (77.3%) were re-evaluated and all (100.0%; 95%CI = 89.7–100.0%) were found to have seroconverted, obviating the need for a second booster dose after study enrollment. The GMT of circulating antibodies in the seronegative group after one booster dose administered subsequent to enrollment increased from 66.9 (95%CI = 56.4–79.3) to 1607.6 (95%CI = 1253.6–2061.7; *p* < 0.0001).

The multivariate logistic regression model showed an association of seropositivity at enrollment with male sex (aOR = 0.4; 95%CI = 0.2–0.7) and with age (in years) at the time of the first dose of varicella vaccine (aOR = 0.84; 95%CI = 0.74–0.95). There were no associations with any of the other determinants (*p* > 0.05; Table 1).

**Table 1:** Determinants of seropositivity at study enrollment according to a multivariate logistic regression model

Determinant	aOR	95%CI	***p***-value
Age at enrollment (years)	0.99	0.82–1.19	0.904
Sex (male vs. female)	0.36	0.28–0.72	0.003
Chronic disease (yes/no)	0.86	0.44–1.68	0.650
Age at the time of the first vaccine dose, basal routine	0.84	0.74–0.95	0.006
Age at the time of the second vaccine dose, basal routine	1.13	0.95–1.33	0.157

Hosmer-Lemeshow Χ^2^ = 5.8; *p* = 0.675

**Fig. 1:**
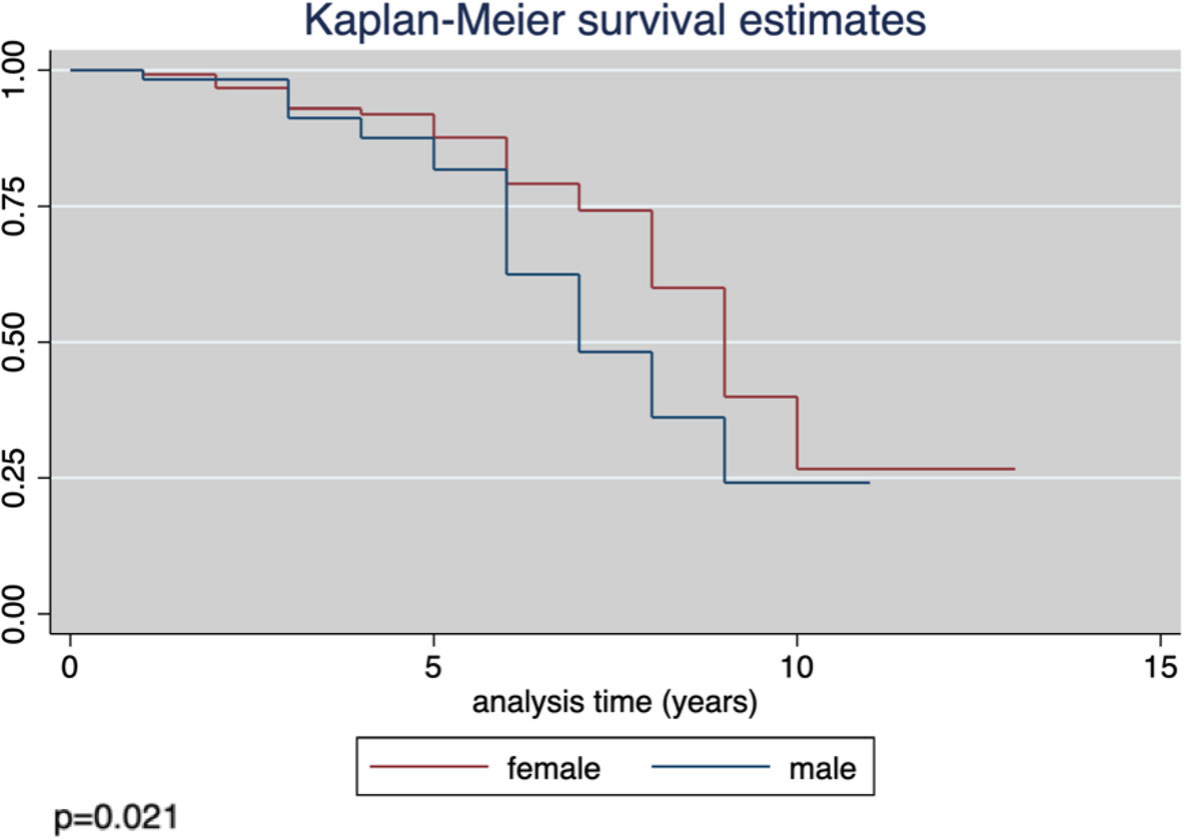
Kaplan-Meier estimates of protective antibody survival (PAS) in males vs. females

The average PAS time was 5.7 ± 2.4 years (range = 1.0–13.0), with an estimated loss of anti-varicella IgG in 50% of the study group after 9 years (95%CI = 8–9). The incidence rate of seronegativity × 100 person-years was 6.0 (95%CI = 4.6–7.6). The difference in the estimated PAS between males and females was significant (*p* = 0.021; Fig. 1), with an incidence rate of seronegativity × 100 person-years of 8.3 (95%CI = 5.8–12.0) and 4.8 (95%CI = 3.4–6.7), respectively. The IRR was 1.75 (95%CI = 1.02–2.97; *p* = 0.031).

According to the multivariate Cox semiparametric regression model, male sex (aHR = 1.73; 95%CI = 1.04–2.88), younger age at enrollment (aHR = 0.71; 95%CI = 0.60–0.83), and older age at the time of the first vaccine dose of the basal routine (aHR = 1.5; 95%CI = 1.2–1.7) were risk factors for PAS and so determinants for loss of circulating antibodies (Table 2).

**Table 2:** Analysis of risk predictors of PAS in a multivariate semiparametric Cox regression model

Predictor	aOR	95%CI	***p***-value
Age at enrollment (years)	0.71	0.60–0.83	< 0.0001
Sex (male vs. female)	1.73	1.04–2.88	0.034
Chronic disease (yes/no)	0.97	0.58–1.62	0.897
Age at the time of the first vaccine dose, basal routine	1.46	1.27–1.67	< 0.0001

Groennesby and Borgan Χ^2^ = 1.9; *p*-value = 0.165

An assessment of the safety of the booster dose based on a 1-month follow-up did not reveal any serious and/or long-term adverse reactions. The most commonly reported reactions were pain at the injection site (9 × 100 subjects), mild fever within 10–15 days after booster administration (3 × 100 subjects), and laterocervical lymphadenopathy (1 × 100 subjects). All of the adverse events regressed after a few days, without sequelae.

## Discussion

Among the > 6000 young adults initially assessed in our study, only 3% had received two doses of the varicella vaccine prior to study enrollment. Since 2003, the National Immunization Plan has supported varicella vaccination for adolescents who are not naturally immunized. Thus, for the population born before 1998, as was the case in the majority of our study participants, varicella coverage in Italy is very low.

Despite two doses of varicella vaccine, almost 34% (95%CI = 27–41) of the enrollees did not have circulating anti-VZV IgG. However, among those who then received a booster dose, an immunity response was elicited such that the seroconversion rate was 100%. Furthermore, the increase in the GMT (from 67 to 1608) after the booster dose resulted in a significantly higher titer than in the group that was seroprotected at enrollment (1608 vs. 657; *p* < 0.0001), indicating a decrease over time in the antibody titer among the vaccinated population.

Our study showed a better immunological response and duration of circulating antibodies in females than in males, based on comparisons of seroprevalence at enrollment, GMT, and PAS, the IRR, and the multivariate regression models. Previous studies of the sex-based differences in the response to vaccines or infection [22, 23] have consistently shown that females have a more effective immune response to immunization, and thus presumably varicella vaccination (immunogenicity and, probably, effectiveness), and against infection.

Our antibody survival model showed that antibody levels tend to decline as early as 1 year after completion of the basal routine, such that after 9 years half of the fully vaccinated population will have lost circulating antibodies. In another study, albeit of low scientific evidence, a period of 10 years was determined [8]. For other live-attenuated virus vaccines, such as the MMR (measles, mumps, rubella) vaccine, the duration of circulating antibodies is more than double that of the varicella vaccine [21, 24, 25]. A loss of circulating antibodies over time was also shown in this study by the multivariate regression models, in which the interval between the last vaccine dose and the serological assessment was identified as a determinant of serosensitivity.

The strengths of this study are that it provides further knowledge on a topic that has been poorly studied thus far, including evidence of sex-based difference in VZV immunity. However, a major limitation was the sample size, although this was not unexpected because, in Italy, varicella vaccination for adolescents who are not naturally immunized became routine only in 2003 whereas the study participants were for the most part born before 1998. Another limitation was our inability to analyze the immune status as a function of vaccine formulation (monovalent vs. MMRV). A determination of the differences between these vaccines is crucial to understanding their immunogenicity performance. In addition, whether the study enrollees had ever come into contact with the wild virus (without developing disease) was unknown. Future research should assess the management of non-responders, which will require a larger sample size and a longer follow-up after routine vaccination (for example, the seroprevalence and GMT should be evaluated at different time point), perhaps also stratifying the study population per vaccine type (monovalent vs. MMRV). This approach will provide a fuller picture of immunogenicity over time.

The efficacy of at least one (and potentially a second) booster dose in fully vaccinated but non-seroprotected healthcare workers (HCWs), such as our study population, has not been well studied. Behrman et al. [26] evaluated the humoral immune responses of 101 HCWs (median age 30 years) who had previously received a two-dose regimen of varicella vaccine. Including 12 (11.9%) who were seronegative at enrollment but received a third vaccine dose during the study. Seven of those 12 HCWs (58.3%) seroconverted after the third dose (safety data were not reported). Our study population comprised a much larger number of serosusceptibles and the seroconversion rate after the booster dose was nearly twice as high. In a 2020 Italian study, Trevisan A et al. [27] tested 234 full vaccinated medical school students and determined a serosusceptibility rate of 21.4%. The authors found a decrease in the antibody titer with an increasing interval from vaccination to the antibody titer evaluation. Within the group of serosusceptible students who received a third vaccine dose, 91.4% seroconverted, without any major symptoms following vaccination. The results of that study were in agreement with our own, especially considering the similar study populations, but Trevisan A et al. [27] found no evidence of a difference between sexes.

The risk of a loss of antibodies in vaccinated individuals over time is an important finding, as between now and the next 10–15 years the loss of circulating antibodies against VZV in the vaccinated population will increase their susceptibility to the virus. Since it is highly unlikely that VZV will be eliminated in the immediate future, the loss of immunity in a substantial portion of the population implies a risk of varicella outbreaks in the coming years. Indeed, many cases of chickenpox among fully vaccinated adults have been reported in the literature, although the disease in this group seems to be less severe [28–30]. However, it should also be noted that the role of cell-mediated immunity in the protection of the non-seroprotected is a matter of debate within the scientific community [26, 31], such that whether serum IgG titers alone accurately reflect vaccine protection is unclear. A 2020 study [32], carried out in a very small sample, compared humoral and T cell immunity in women of childbearing age who had been immunized against VZV either by vaccination or naturally. The authors found no significant differences in the antibody titers between the two groups (*p* > 0.050), although the naturally immunized group had significantly higher levels of VZV antigen-specific CD4 T cells (*p* = 0.004).

## Conclusions

Finally, while further research is needed, the results of our study have important implications for HCWs, as the inclusion of a screening model in the routine assessment of their biological risk may prevent nosocomial varicella infections, especially in high-risk settings (pediatric wards, gynecology units, infectious disease departments, etc.). Moreover, as chickenpox can be particularly severe in adults, HCWs should be protected by varicella vaccination, which will also confer protection from HZ [33].

In summary, the time between varicella vaccination and the antibody titer evaluation is a determinant of a decline in serum levels of circulating antibodies against VZV and thus of immunity to the virus. Among the fully vaccinated but non-seroprotected, the administration of a third booster dose is effective and safe in achieving seroconversion. These results are an important contribution to public health planning.

## Data Availability

The datasets generated and/or analysed during the current study are not publicly available due to sensible contents but are available from the corresponding author on reasonable request.

## Abbreviations

CDC: Center for Disease Control and Prevention
WHO: World Health Organization
VZV: Varicella zoster virus
MMRV: Measles, mumps, rubella, varicella
HCWs: Healthcare workers
HZ: Herpes zoster
GIAVA: Regional Immunization Database
